# Nano-Sized Fe(III) Oxide Particles Starting from an Innovative and Eco-Friendly Synthesis Method

**DOI:** 10.3390/nano10020323

**Published:** 2020-02-14

**Authors:** Ludovico Macera, Giuliana Taglieri, Valeria Daniele, Maurizio Passacantando, Franco D’Orazio

**Affiliations:** 1 Department of Industrial and Information Engineering and Economics, University of L’Aquila, Piazzale E. Pontieri 1, 67100, Monteluco di Roio, Roio Poggio, I-67100 L’Aquila (AQ), Italy; ludovico.macera@graduate.univaq.it (L.M.); valeria.daniele@univaq.it (V.D.); 2 Department of Physical and Chemical Sciences, University of L’Aquila, via Vetoio, I-67100 L’Aquila (AQ), Italy; maurizio.passacantando@aquila.infn.it (M.P.); franco.dorazio@aquila.infn.it (F.D.)

**Keywords:** nanoparticles synthesis, iron oxide nanoparticles, 2-line ferrihydrite, hematite nanoparticles, magnetic measurement, XPS, XRD, TEM, FE-SEM

## Abstract

This paper introduces an original, eco-friendly and scalable method to synthesize ferrihydrite nanoparticles in aqueous suspensions, which can also be used as a precursor to produce α-hematite nanoparticles. The method, never used before to synthesize iron oxides, is based on an ion exchange process allowing to operate in one-step, with reduced times, at room temperature and ambient pressure, and using cheap or renewable reagents. The influence of reagent concentrations and time of the process on the ferrihydrite features is considered. The transformation to hematite is then analyzed and discussed in relation to different procedures: (1) A natural aging in the water at room temperature; and (2) heat treatments at different temperatures and times. Structural and morphological features of the obtained nanoparticles are investigated by means of several techniques, such as X-ray diffraction, X-ray photoelectron spectroscopy, attenuated total reflectance Fourier transform infrared spectroscopy, transmission and scanning electron microscopy, thermal analysis, nitrogen adsorption and magnetic measurements. Ferrihydrite shows the typical spherical morphology and a very high specific surface area of 420 m^2^/g. Rhombohedral or plate-like hexagonal hematite nanoparticles are obtained by the two procedures, characterized by dimensions of 50 nm and 30 nm, respectively, and a specific surface area up to 57 m^2^/g, which is among the highest values reported in the literature for hematite NPs.

## 1. Introduction

Iron oxide is polymorphic in nature, including sixteen compounds between oxides, hydroxides and oxy-hydroxides. In most of them, iron is present in the trivalent state, such as in hematite (α-Fe_2_O_3_), the more stable polymorph occurring in nature, and in ferrihydrite, (Fe_5_HO_8_·4H_2_O), a naturally nanoscale amorphous iron oxy-hydroxide mineral, present in most biological and geochemical environments [1]. The α-Fe_2_O_3_ is structurally characterized by a hexagonal unit cell, in which two-thirds of the octahedral sites are occupied by Fe^3+^ ions (corundum structure) [2], and orders antiferromagnetically below its Nèel temperature, T_N_ ≈ 955 K. However, at room temperature, it presents a week magnetization, due to slight canting of the magnetic moment with respect to the antiferromagnetic order. It is an n-type semiconductor (Eg = 2.1 eV), and it has been considered for a wide range of applications because it is readily available, non-toxic and an environment-friendly material, hardly affected by changes in oxidation [3,4,5]. If produced in the form of nanoparticles, due to the low toxicity, stability and unique properties, hematite can be considered ideal for applications in a wide range of emerging fields. In particular, nowadays it represents a low-cost photocatalyst for water oxidation, but also an interesting opportunity for artificial photosynthesis, pigments, gas sensors, catalysts, magnetism, lithium ion batteries, electrochemical capacitors and biomedical applications [6,7,8,9,10,11,12,13,14]. Hematite nanoparticles can be synthesized by means of several methods, from physical, chemical, or biological routes. However, taking into account the simplicity, the costs and the reproducibility, better results are obtained by chemical routes, including chemical precipitation, sol-gel, hydrothermal, surfactant mediated precipitation, emulsion precipitation, microemulsion precipitation, electro-deposition, and microwave-assisted hydrothermal technique [4,13,14,15,16]. In addition, many of these routes are based on the use of ferrihydrite as precursor [17,18,19,20,21,22]. 

For its significant role as a precursor for hematite nanoparticles, as well as for its intrinsic properties, a widespread interest in the methods to synthesize ferrihydrite is ever growing. Actually, ferrihydrite is a biocompatible material, characterized by a high reactivity and high ion adsorption capacity, paramagnetic properties at room temperature, as well as by tunable band gap between 1.3 and 2.5 eV [1,23]. These properties lead to diffuse applicability in several fields, such as in magnetic applications, wastewater treatments, metallurgical industry, lithium ion batteries, or in agricultural germination and growth of maize [24,25,26,27,28]. The methods of synthesis are typically based on chemical precipitation routes [8,23,24,25], but they require extra purification steps and long times to obtain the final product, so that the possibility to produce large amounts of ferrihydrite nanoparticles (NPs) with high yields is still a challenge.

Aim of this paper is to obtain ferrihydrite NPs by means of an original, one-step, environmental-friendly and scalable synthesis and to use them as precursors for the production of hematite NPs as well. The innovative synthetic procedure represents the focal point of this work. It is based on an ion exchange process occurring at room temperature and ambient pressure, between an anion exchange resin (OH form) and an iron(III) chloride aqueous solution. In proper conditions, the anion resin exchanges its hydroxyl groups with chloride ions, leading to high supersaturation conditions and to burst nucleation of the solid phase, synthesizing the desired hydrous ferric oxide NPs dispersed in water, with no other secondary products. At the end of the synthesis, the resin is separated from the obtained product through a simple sieving procedure, then it can be regenerated in a few minutes with a solution of NaOH 1M and used again in a new synthesis. The possibility to regenerate the resin several times and the operating conditions limit the energy consumption, the wastes and consequently the emissions of the process, guaranteeing a low environmental impact. In comparison with the coprecipitation methods, requiring several purifications steps to obtain a pure product, such as washing or dialysis, the here proposed ion exchange process allows to directly obtain a pure product, promising a better performance given its reduced time and higher yield. The one step and cyclic procedure of this new method can be promising for an easily scalable production.

Considering that the particle size distribution is affected by the nucleation step, as well as by the successive growing mechanisms, we investigated the feature of the produced NPs in relation to the supersaturation conditions and to the duration time of synthesis. Then, the ferrihydrite NPs with the best features in terms of surface area are used as a precursor to obtaining the hematite nanoparticles. In particular, although the transformation from ferrihydrite to hematite has been studied for many years, the mechanisms at the base of the formation of hematite from ferrihydrite are still debated in recent literature, and this issue is indeed still current [8,17,29]. For this reason, the transformation of the ferrihydrite, obtained by this new method, to hematite NPs is investigated as well. Two procedures are followed: The first one consists of the aging, at room temperature, of the ferrihydrite NPs in aqueous suspension up to seven months. In the second procedure, the hematite NPs are obtained after calcinating the initial ferrihydrite suspension, analyzing the influence of time and temperature of the calcination process on the NPs features. The structural and morphological features of the produced NPs are investigated by several techniques, such as X-ray diffraction (XRD), X-ray photoelectron spectroscopy (XPS), transmission and scansion electron microscopy (TEM, SEM), attenuated total reflectance Fourier transform infrared spectroscopy (ATR-FTIR), thermal analysis (TG-DTA) and surface area measurements using the Brunauer–Emmett–Teller (BET) method. In addition, magnetic measurements of ferrihydrite and hematite NPs, both after the aging and after the calcination procedure, are carried out at room temperature, in order to characterize their magnetic properties.

## 2. Materials and Methods

### 2.1. Materials

Iron chloride hexahydrate (FeCl_3_-6H_2_O) is supplied by Zeus (99% purity); ion-exchange resin Dowex Monosphere 550A is supplied by Sigma Aldrich (St. Louis, MO, USA.), in the form of translucent spherical beads characterized by a particle size equal to 590 ± 50 µm.

### 2.2. Synthesis of Ferrihydrite Nanoparticles by Ion Exchange Process

In order to prepare ferrihydrite NPs, a proper amount of the anionic resin, characterized by hydroxyl groups on its substrate (R-OH), is mixed with an aqueous solution of iron(III) chloride, working at room temperature (20 °C), under moderate stirring, and maintaining them in contact up to 60 min. After this period, the obtained NPs are separated from the resin through a simple sieving procedure (mesh 180 µm). When mixed together, the substitution of -OH groups on the resin substrate with chlorides ions (Cl^−^) in solution can lead, in conditions of supersaturation, to the formation of iron(III) hydroxide (Fe(OH)_3_) with no other secondary phase, according to the following reaction [30]:

FeCl_3_ + 3R-OH → Fe(OH)_3_ + 3R-Cl. (1)

However, at OH/Fe ratios >0.5, the hydrolysis of ferric solutions initially determines the formation of mono and binuclear species, which interact to produce polynuclear species [21]. These species grow up to an amorphous ferric oxide hydrate precipitate, called ferrihydrite (from here called Fh) [31]. Actually, in these conditions, Fh is considered the first stable product of ferric precursor hydrolysis in water, having a variable composition especially with respect to OH and H_2_O. This variability generates a wide range of chemical compositions and formula, not univocally defined, due to the difficulty of distinguishing between structural OH, structural H_2_O and physically adsorbed H_2_O. In the literature, many formulas, such as Fe_5_HO_8_·4H_2_O, 5Fe_2_O_3_·9H_2_O, FeOOH⋅0.4H_2_O, Fe(OH)_3_·nH_2_O, are reported [1,7]. Therefore, due to the instability of Fe(OH)_3_ in water, the precipitated phase formed after the ion exchange process is expected to be Fh, and the reaction (1) can be substituted by:

FeCl_3_ + 3R-OH → Fh + 3R-Cl (2)

In order to follow the kinetics of the ion exchange process, during the stirring operations, we measured the chloride concentration (CC) in samples taken at different times (t), from the beginning until the end of the reaction (t = 0, 0.5, 1, 3, 5, 15, 30, 60, minutes, respectively). The CC values are measured by means of an ion sensitive electrode for Cl (Methrom). Moreover, since from reactions (1) and (2) a reduction in the chloride concentration can be related to the Fh formation, we estimated the yield of the production (Y) at different synthesis times according to the following formula:

(3) $$Y\left(t\right)=\left(\frac{{\mathrm{CC}}_{0}-\mathrm{CC}}{{\mathrm{CC}}_{0}}\right)*100$$

where CC_0_ is the initial chloride concentration.

The influence of the supersaturation conditions on the NPs features is investigated too, considering different concentrations of the aqueous ferric chloride solutions, equal to 0.1 M, 0.5 M and 1 M. The obtained suspensions are called Fh_0.1M_, Fh_0.5M_ and Fh_1M_, respectively. 

Finally, considering the fast kinetics of the ion exchange process, (as also observed in our previous works [32,33,34,35]), the Fh_1M_ sample separated from the resin in just 3 min, is analyzed; at this time a drastic reduction in the chloride concentration is measured. This sample is called Fh_1Mfast_.

### 2.3. Production of Hematite Nanoparticles from Ferrihydrite as Precursor

As already observed in the literature [17,18,19,20,21], ferrihydrite is a metastable iron compound, and it can be transformed into hematite, goethite or akageneite, depending on temperature, pH and composition. The transformation of ferrihydrite into hematite can occur in water, at neutral pH, at room temperature. However, in these conditions it requires very long times to complete (up to 12 years), revealing after one year a transformation not more than 40%. The transformation in water can be accelerated by increasing the temperature, but it remains incomplete anyway. In order to have a complete and fast transformation, dry heating at high temperature is needed (calcination process).

In this paper, the transformation from the obtained ferrihydrite to hematite is analyzed considering only the sample characterized by the highest BET value, which is the Fh_1M_ sample, as will be shown below. In particular, the production of hematite is carried out following two procedures. In the first procedure, the aqueous suspension Fh_1M_ is maintained at room temperature (RT) up to seven months of aging, analyzing the phase transformation by XRD, at different times of aging (after 1, 1.5, 2, 3, 5, 7 months). Two representative samples are chosen to investigate structural, morphological and magnetic features, after two months and seven months of aging, named H_RT2_ and H_RT7_, respectively. The second procedure consists in the calcination of ferrihydrite powder obtained after a lyophilization of the suspension Fh_1M_. The heat treatments are carried out at different temperatures, from 100 °C to 700 °C, with steps of 50 °C, and times ranging from 2 to 24 h. For each sample, the phase transformation is followed by XRD. A representative sample is chosen to investigate structural, morphological and magnetic features, that is the sample obtained at 500 °C for 3 h (the lowest conditions calcination needed to obtain pure hematite), named H_500_.

### 2.4. Characterization of the Nanoparticles

The phase composition and crystallinity of both ferrihydrite and hematite NPs are investigated by X-ray diffraction (XRD). XRD scans are recorded at room temperature on a PANalytical X’Pert PRO apparatus using Cu-Kα radiation, equipped with a monochromator. Concerning the ferrihydrite samples, as well as the samples aged at room temperature in water, 0.12 mL of each suspension are homogeneously taken, then deposited on a zero-background sample holder and dried under nitrogen. For the calcined samples, measurements are performed on the dry powders, sieved on a zero-background holder. In both cases, XRD patterns are recorded by a step scan in the range 2θ from 3° to 80°. Each experimental diffraction pattern is elaborated by a profile fit software, High Score Plus (PANalytical, Almelo, the Netherlands), and crystalline phases are attributed by ICDD and ICSD reference databases. XRD peak broadening analysis is performed, in order to evaluate the average crystallite size, D_hkl_, (i.e., coherent X-ray scattering domains). For this task, the well-known Debye-Scherrer formula is used [36]. The elemental composition, the chemical state and the electronic state of the elements that are present in the samples are investigated by X-ray photoelectron spectroscopy (XPS). XPS measurements are recorded at room temperature using a PHI 1257 system equipped with an Mg X-ray source (hν = 1253.6 eV) with a hemispherical analyzer. Transmission electron microscope (TEM), working with a 100 kV acceleration voltage, CM100 (Philips, Amsterdam, the Netherlands) and field emission scanning electron microscope GeminiSEM 500 (ZEISS, Oberkochen, Germany) are used to observe morphology and dimensions of NPs or of their aggregates. For these observations, samples are prepared by dropping the synthesized colloidal suspensions, previously diluted in deionized water, onto suitable TEM grids or onto SEM stabs. In particular, the samples are deposited with a concentration of 0.01 g/L on the grids and 10 g/L on the stabs. Regarding TEM images, the particles size distribution is evaluated by using ImageJ software. Attenuated total reflectance-Fourier transform infrared (ATR-FTIR) spectroscopy measurements are performed, by a Thermo Nicolet Nexus spectrophotometer; data are collected from 400 to 4000 cm^−1^. Thermal analyses are carried out in air at a heating rate of 10 °C/min, using a LINSEIS L81 apparatus. Nitrogen adsorption measurements are performed at 77 K, using the ASAP 2000 system (Micromeritics, Norcross, GA, USA), utilizing Brunauer Emmett-Teller (BET) calculations for surface area. About 0.2 g of dry powders are first outgassed for about 16 h at 150 °C (5·10^−3^ Torr). The pore-size distribution is determined from the desorption branch of the isotherms using the BJH (Barett–Joyner–Halenda) method. Finally, magnetic characterization was performed using a PMC Micromag 2900 alternated gradient magnetometer (AGM) (Lake Shore Cryotronics, Westerville, OH, USA).

Concerning the preparation of the samples, XPS, ATR-FTIR, thermal analyses, nitrogen adsorption and magnetic measurements, the samples are analyzed in the form of powders, obtained after lyophilization of the initial suspensions.

## 3. Results and Discussion

### 3.1. Production and Characterization of Ferrihydrite NPs

For all the synthesis, the kinetic of the ion exchange process is reported in Table 1 and in Figure S1, respectively. The obtained results denote a very fast kinetic, giving rise to a production yield Y of more than 99% in the first minute, the value that even increases with increasing the reagent concentration. Moreover, independently from the initial chloride concentration, at the end of the synthesis, a residual CC value of about 20 mg/L is measured. In Table 1, we can also observe that pH rapidly changed from acid values, related to the initial iron(III) chloride solutions, to neutral values after 3 min from the beginning of the synthesis.

These measurements confirm that, for all reagent concentrations, the substitution on the resin substrate of -OH groups with chlorides ions (Cl-) is extremely fast, leading to a very high reduction in the chloride content within 3 min, corresponding to a yield of production (Y) up to 99.9%. 

**Figure 1:**
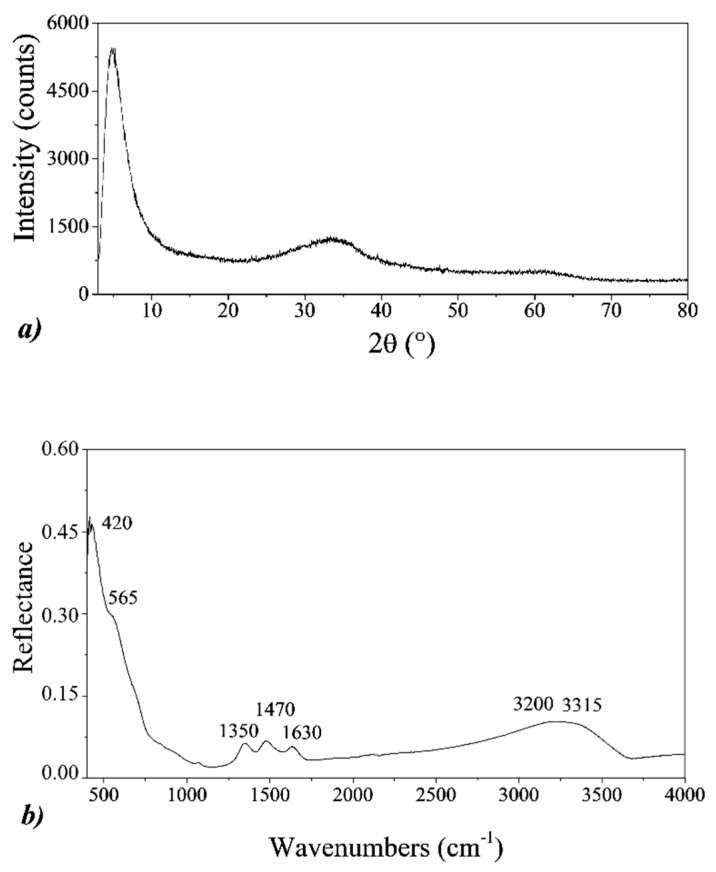
(**a**) XRD pattern and (**b**) attenuated total reflectance Fourier transform infrared spectroscopy (ATR-FTIR) spectrum of Fh_1M_ sample.

From XRD analyses, all the obtained samples appear amorphous, characterized by two broad halos, at 2θ = 34° and 2θ = 61°, respectively, as shown in Figure 1a. These halos can be attributable to pure 2-line ferrihydrite [24], the lowest crystalline form of hydrous ferric oxide, typically obtained by fast hydrolysis near pH 7 (OH/Fe ~ 3) [21]. Moreover, in all the XRD patterns, a strong reflection at around 2θ = 5° is observed, which corresponds to a d-spacing of about 1.8 nm. This reflection is not observed for the empty sample holder or for other samples, such as pure hematite (reported in Section 3.2). For these reasons, it can be associated with an ordered mesoporous structure of the 2-line ferrihydrite [37], as well as to the presence of a uniform nanoparticle size [38].

ATR-FTIR measurements confirm the formation of the 2-line ferrihydrite, giving the same results for all the samples. As an example, in Figure 1b, the reflectance spectrum of Fh_1M_ sample is shown. The large bands at around 3250 cm^−1^ are attributed to the broad O-H stretching vibration, related to the structural hydroxide of the 2-line ferrihydrite at 3315 cm^−1^, as well as from adsorbed H_2_O at 3200 cm^−1^. The small reflection band at 1630 cm^−1^ is related to the O-H bending vibration from H_2_O, while the strong and broad bands at 565 and 420 cm^−1^ are typical of the low crystalline ferrihydrite and are attributed to the Fe-O-Fe stretching vibrations. Moreover, the asymmetric and symmetric C-O stretching modes at 1470 cm^−1^ and 1350 cm^−1^, respectively, are observed. These bands are typically found in 2-line ferrihydrite samples, due to the carbonate and/or bicarbonate absorbed from water and to the CO_2_ from the air as well [39,40,41].

**Figure 2:**
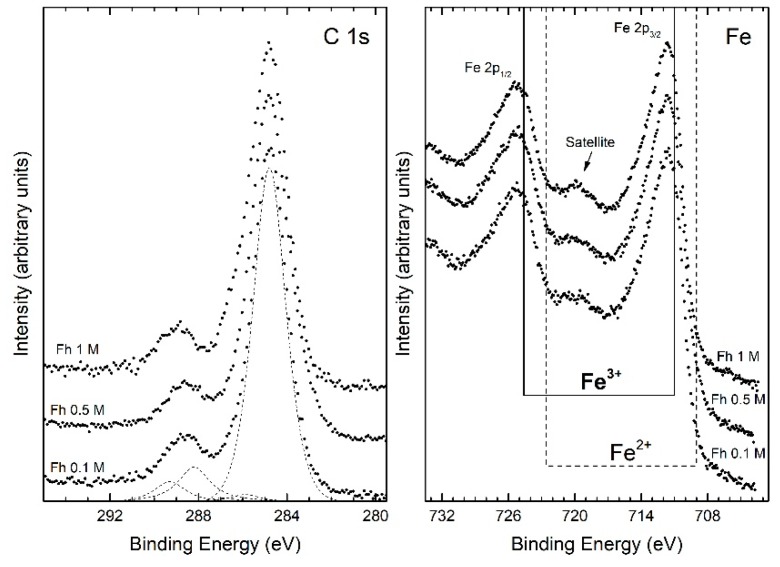
Core level C 1s and Fe 2p XPS spectra of samples at different initial chloride concentrations: 0.1 M, 0.5 M, and 1 M.

XPS survey spectra, for the ferrihydrite samples, reveal the presence of Fe, O and C as the only elements, confirming that the starting reagent has been completely used up, and no contamination species were observed within the sensitivity of the technique. The binding energy (BE) calibration of the spectra has been referred to as carbon 1s peak located at BE = 284.8 eV. Figure 2 shows the C 1s and Fe 2p electron core-level XPS spectra acquired from the ferrihydrite samples as a function of the initial chloride concentration.

The C 1s spectra are typical of the adventitious carbon layer, due to atmosphere exposition (CO_2_), characterized by the main peak around 285 eV. In particular, the peaks are all nicely fitted by the sum of five components assigned to C atoms belonging to: C–C (284.8 eV), C–OH (285.9 eV), C–O–C (286.9 eV), C=O (288.2 eV), C=O(OH) (289.3 eV) [42,43,44]. The XPS peaks of the Fe 2p_3/2_ and 2p_1/2_, for the same samples at different initial chloride concentrations, are also shown in Figure 2. A characteristic satellite peak is located approximately 8 eV higher than the main Fe 2p_3/2_ peak [45,46]. The binding energies of Fe 2p_3/2_ and Fe 2p_1/2_ obtained from the present study are 711.4 and 725.0 eV, respectively. The satellite peak obtained at 719.6 eV is clearly distinguishable and does not overlap with the Fe 2p_3/2_ or Fe 2p_1/2_ peaks. From these results, we can consider that each sample has an oxidation state like Fe^+3^, as also shown in Figure 2 with the dotted and continuous lines indicating the position in the binding energy of the 2p_3/2_ and 2p_1/2_ of iron peaks for the oxidation state Fe^2+^ and Fe^3+^, respectively.

As concerns thermogravimetric and differential thermal analyses (TG-DTA), similar results are obtained for Fh_0.1M_, Fh_0.5M_, Fh_1M_ and Fh_1Mfast_ samples. In Figure S2, the representative curves measured for Fh_1M_ sample are reported. From TGA a whole weight variation of about 15% is measured, consistent with the dehydration of ferrihydrite towards hematite. However, the highest weight loss (12%) is observed up to 250 °C, corresponding to about 80% of the whole weight loss during the analysis. Moreover, in accordance with literature references on 2-line ferrihydrite, the DTA curve shows an endothermic peak, at about 100 °C, and two exothermic peaks, at about and 230 °C and 280 °C, due to the dehydration of ferrihydrite [47].

**Figure 3:**
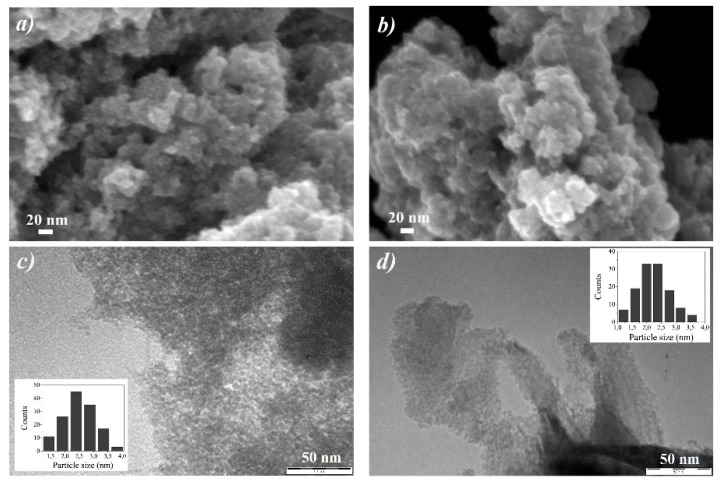
Ferrihydrite NPs from diluted suspensions (0.01 g/L) prepared on TEM suitable grids. FE-SEM images of: (**a**) Fh_0.1M_, (**b**) Fh_1M_. TEM images of: (**c**) Fh_0.1M_, (**d**) Fh_1M_. Particles size distributions (insets) carried out on a population of ca. 130 NPs.

FESEM images, performed on typical stabs prepared by deposition of drops at a concentration of 10 g/L, reported in Figure S3, displayed similar results for all the ferrihydrite suspensions. In particular, we observed that all the samples are composed by a superimposition of pseudo-spherical particles of about 10 nm or less, forming several aggregates. As concerns the ferrihydrite samples examined as diluted suspensions (0.01 g/L) on TEM grids, the results are similar for Fh_0.1M_ and Fh_0.5M_ samples and for Fh_1M_ and Fh_1Mfast_ samples, respectively. In Figure 3, the representative FESEM and TEM images obtained for Fh_0.1M_ and Fh_1M_ samples are reported.

FESEM images show that both samples are always constituted by disordered assemblies of very small particles (<5 nm), which form large mesoporous aggregates without a definite shape (Figure 3a,b). TEM images emphasize that both samples consist of a large number of nanoparticles, having dimensions in the range 1–5 nm (Figure 3c,d), aggregated to form large and dense agglomerates typical of 2-line ferrihydrite features [48]. Many mesopores are also visible, with diameters of few nanometers. TEM image related to Fh_1M_ sample shows that, increasing the reagent concentrations, a more compact mesoporous aggregation occurs (Figure 3d), probably due to the presence of slightly smaller nanoparticles dimensions, evidenced by the particles size distribution analysis (see the inset of Figure 3d). The difference observed in the groups of samples can be attributed to the different nucleation occurring during the synthesis. Actually, at the beginning of the synthesis, a fast and diffuse nucleation occurs and all solid primary NPs form at the same time and under the same conditions in all the volume of reaction (that is, around each particle of the resin). In addition, due to the very rapid kinetic of the ion exchange process and to the low solubility of ferrihydrite [49], a fast depletion of the OH^-^ sites on the resin substrate occurs, hindering the growth of such NPs as well. For these reasons, a quite uniform size distribution of the ferrihydrite NPs is obtained, as revealed by the experimental size distribution analyses. Moreover, the nucleation is enhanced for Fh_1M_ and Fh_1Mfast_ samples because of the higher concentration of the initial reagents, leading to a higher degree of supersaturation and to a greater nucleation rate, resulting in relatively smaller nanoparticles.

**Figure 4:**
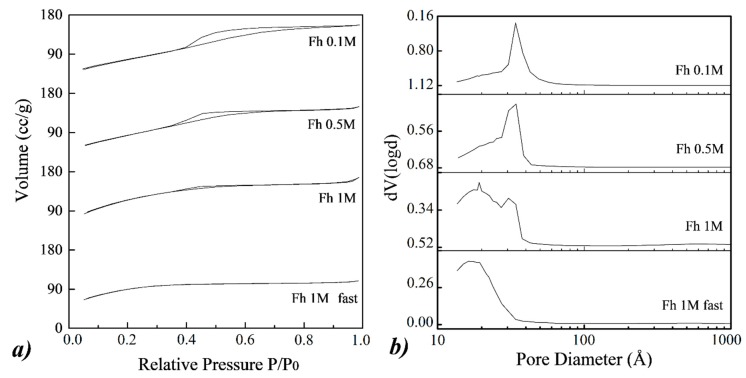
(**a**) N_2_ adsorption/desorption isotherms and (**b)** Barett–Joyner–Halenda (BJH) distributions for the desorption branch of the isotherms of different ferrihydrite samples. From top to bottom: Fh_0.1M_, Fh_0.5M_, Fh_1M_, and Fh_1Mfast_.

This hypothesis is confirmed by the nitrogen adsorption measurements, reported in Figure 4. The isotherms of Fh_0.1M_ and Fh_0.5M_ samples can be related to isotherms of type IV, with the typical H1 hysteresis loop of mesoporous materials, which is associated with capillary condensation taking place in mesopores, according to the IUPAC classification [50]. In the case of Fh_1M_ sample, the profile of the isotherm can be more related to type I(b), characteristic of materials having pores with diameters in the upper range of the classical micropore domain, containing both wider micropores and narrow mesopores (<2.5 nm) [22,51]. The tiny adsorption-desorption hysteresis in the interval 0.30 < P/P_0_ < 0.60 in the isotherm of Fh_1M_ highlights the presence of small mesopores.

The differences between the three samples are confirmed by the BJH pore size distributions, shown in Figure 4b). Actually, Fh_0.1M_ and Fh_0.5M_ samples exhibit pore size distributions mainly peaked in the range 3–4 nm, while Fh_1M_ sample presents a bimodal distribution of pores, peaked at 1–2 nm and 3–4 nm. The influence of the reagent concentrations (from 0.1 M to 1 M) on the obtained 2-line ferrihydrite NPs is also marked by the BET and BJH calculations, shown in Table 2. If the reagent concentration is increased from Fh_0.1M_ to Fh_1M_, a reduction in the BJH pore diameter, from 3.4 nm to 1.5 nm, respectively, is found. Correspondingly, the BET surface area clearly increases from 283 m^2^/g to 421 m^2^/g. In particular, the latter value, relative to the Fh_1M_ sample, is considerably higher than most of the experimental results for ferrihydrite reported in previous literature [52].

Regarding the 1M sample obtained after 3 min, Fh_1Mfast_, the profile of the isotherm is attributable to a type I(a), characteristic of microporous materials, having pores below 2 nm and no adsorption-desorption hysteresis. In fact, the BJH pore size distribution of Fh_1Mfast_ is peaked in the range 1–2 nm, and from Table 2, an average BJH pore diameter of 1.5 nm is observed as well. Correspondingly, a BET value of 327 m^2^/g is measured, lower than Fh_1M_ sample. This relatively low value could be probably due to the resolution limit of the instrument (>10 Å) or to the limitations often related to monolayers [53,54]. For these reasons, a higher BET value for the Fh_1Mfast_ sample would be conceivable.

**Figure 5:**
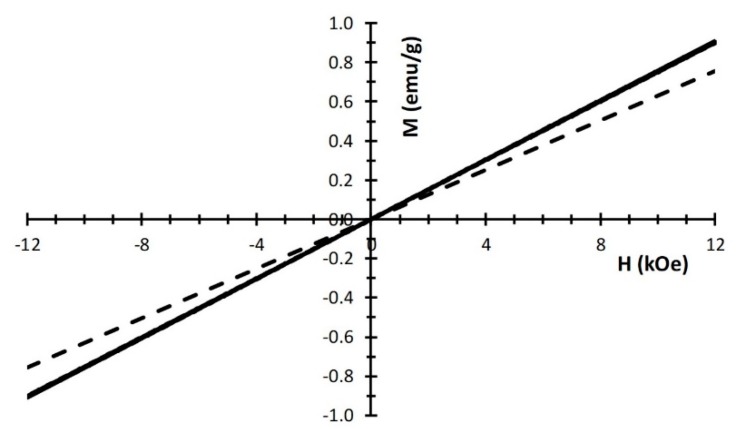
Room temperature magnetization data of different ferrihydrite samples: Fh_0.1M_ (dotted line), Fh_0.5M_, Fh_1M_, and Fh_1Mfas_t (continuous lines, overlapping each other). The linear dependence is observed up to the maximum applied field (12 kOe).

In Figure 5, the magnetization response at room temperature for all the ferrihydrite samples is showed. The linear magnetic field dependence is consistent with the paramagnetic behavior found by most of the authors at ambient temperature, where also a contribution from antiferromagnetic susceptibility must be taken into account [23,55,56]. On the contrary, Pannalal et al. [57] found a small hysteresis and departures from linearity in the initial part of the M(H) curve. However, these discrepancies are common for ferrihydrite compounds, due to the peculiar compositional complexity and the varieties of the nanostructures of the aggregates. Porosity and particle dimensions determine the surface to volume ratio, and as a consequence, they influence the magnetic character, which is strongly affected by the nanoscale and surface conditions of the compound. In this respect, the slight increase of the magnetic susceptibility with increasing reagent concentration, shown in Figure 5, may be ascribed to the higher degree of aggregation and a consequent increase of interparticle interaction (confirming what discussed above), leading to a shift of the distribution of magnetic moments towards higher values [56]. In particular, the magnetic susceptibilities are found to be 6.29 × 10^−5^ emu/(g∙Oe) for Fh_0.1M_, 7.51 × 10^−5^ emu/(g∙Oe) for Fh_0.5M_, 7.54 × 10^−5^ emu/(g∙Oe) for Fh_1M_ and 7.58 × 10^−5^ emu/(g∙Oe) for Fh_1Mfast_. Assuming Curie-like temperature dependence, these values correspond to about 2.5 μ_B_ for Fh_0.1M_ and 3.0 μ_B_ for the other three samples: In all cases less than the theoretical value (5.9 μ_B_) for isolated Fe^3+^ ions.

### 3.2. Production and Characterization of Hematite NPs

**Figure 6:**
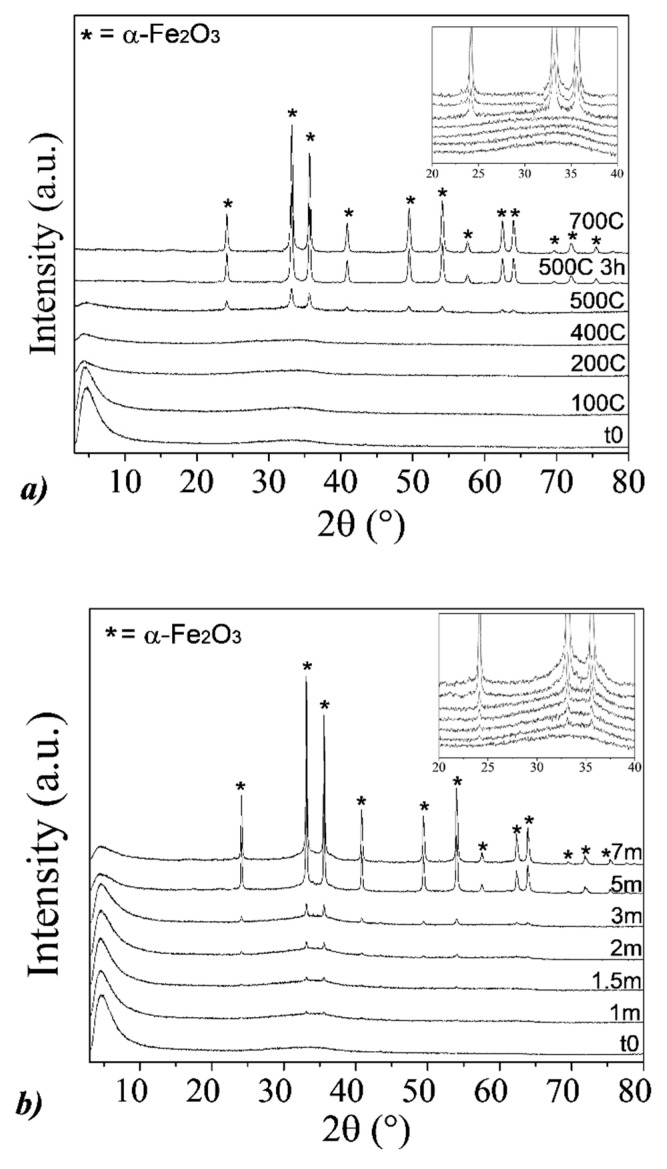
XRD patterns of Fh_1M_ samples: (**a**) At different aging times (t_0_ = fresh ferrihydrite, m = months) at room temperature; (**b**) at different calcination temperatures after 2 h (except for the sample calcined at 500 °C, where the result for 3 h is reported too). In the insets: Enlargement of the 20–40° range.

XRD results of the Fh_1M_ samples, aged at RT up to seven months and calcined at different temperatures, are reported in Figure 6. For the samples obtained after the calcination process, only the results after 2 h are shown, considering that no relevant variations between 2 or 24 h are observed; exception is the samples calcinated at 500 °C, for which the result obtained after 3 h is reported as well.

The XRD patterns referred to the samples aged at RT, at different times (Figure 6a), show a gradual conversion of the ferrihydrite into hematite, by increasing the aging time. In particular, after the first month, we observed the formation of the main Bragg peaks attributable to the hexagonal crystalline structure of α-hematite (α-Fe_2_O_3_, ICSD #98-016-1292), that proceeds up to seven months where α-hematite represents the main observable phase, characterized by an average crystallite size of about 50 nm. In parallel to the formation of hematite, the progressive transformation of ferrihydrite is shown, having its main broad halo at 2θ = 34° which tends to show a more defined profile with time (see the inset). In addition, with increasing the aging time, a progressive decrease of the peak at 5° can be clearly observed, resulting not completely absent after seven months of aging where a residual content of ferrihydrite is still revealed. Concerning the XRD patterns at different calcination temperatures (Figure 6b), the formation of the hexagonal crystalline structure of α-Fe_2_O_3_ is observed only at 500 °C, but not at lower temperatures. In particular, after calcination at 500 °C for 3 h, the broad halos completely disappear, indicating that the conversion of ferrihydrite into hematite can be considered complete. As regards X-rays broadening analysis, the hematite phase is here characterized by an average crystallite size of about 30 nm. In addition, the peak at 5° drastically reduces at 200 °C, where we measured the most dehydration of ferrihydrite in DTA analysis. Considering that this peak can be associated with an ordered mesoporous structure of the 2-line ferrihydrite and that the unique, recognizable phase at temperatures lower than 500 °C is ferrihydrite, the significant reduction of the 5° peak can be related only to a strong aggregation of the ferrihydrite NPs.

The differences arising from the two procedures, both in the formation of hematite and in the reduction of the peak at 5°, underline different mechanisms of phase transformation from ferrihydrite into hematite [21]. Actually, during the aging in water, the transformation occurs by a gradual oriented attachment of the ferrihydrite NPs that helps or is even necessary to trigger crystallization. During such gradual oriented attachment, the α-Fe_2_O_3_ crystallization can occur by a topotactic transformation at solid phase, through dehydration of ferrihydrite and reorientation of iron ions favored, underwater, also at room temperature [8,17,19]. On the contrary, in dry heating conditions, with increasing temperature, ferrihydrite NPs tend to dehydrate and aggregate, but the reorientation of iron ions, and consequently the formation of α-Fe_2_O_3_, is limited, due to the restricted mobility of ions and it can occur only at high temperatures (i.e., 500 °C).

The ATR-FTIR analyses, performed on the H_RT2_, H_RT7_ and H_500_ samples, are reported in Figure S4. The results confirm that in water, by increasing the aging time, ferrihydrite gradually and partially converts into hematite, whereas, following the calcination process, ferrihydrite transforms into pure hematite at temperatures ≥500 °C. The bands attributed to hematite are the typical Fe-O stretching vibrations at about 520 and 440 cm^−1^. In particular, we observed that, for H_RT2_ and H_RT7_ samples, the Fe-O bands become more defined with increasing the aging time. Conversely, in H_500_ sample these bands result well defined and the only ones present.

**Figure 7:**
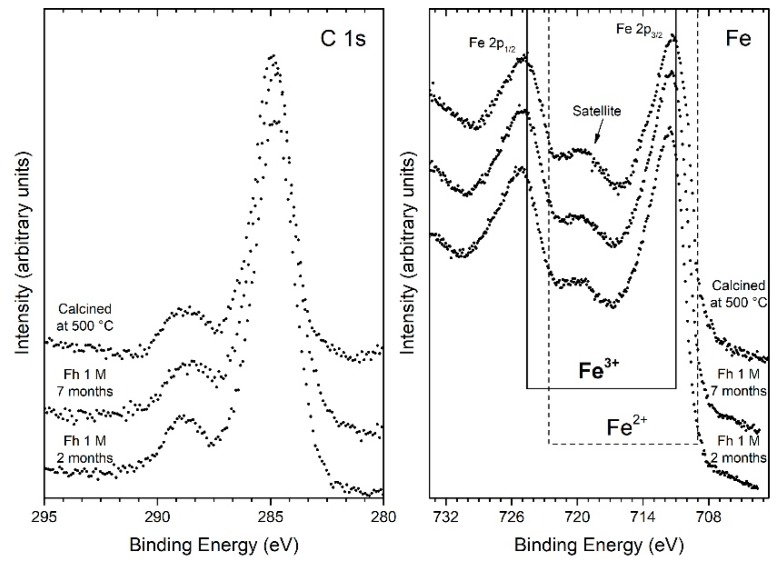
Core level C 1s and Fe 2p XPS spectra of the samples of hematite aged two months at RT (H_RT2_), aged seven months at RT (H_RT7_), and calcined at 500 °C (H_500_).

XPS measurements for the hematite samples obtained from the two procedures, reported in Figure 7, show the presence of Fe^3+^ oxidation state, underlining the existence of hematite or ferrihydrite. Moreover, for the samples H_RT7_ and H_500_, a slight shifting of the Fe 2p_2/3_ peak towards lower binding energy is observed, in accordance with the typical signal related to the prevalence of hematite with respect to other oxy-hydroxy Fe(III) compounds [58]. As concerns the C1s signals, similarly to what observed for the ferrihydrite samples, the results are attributable to the presence of the adventitious carbon contamination (CO_2_).

**Figure 8:**
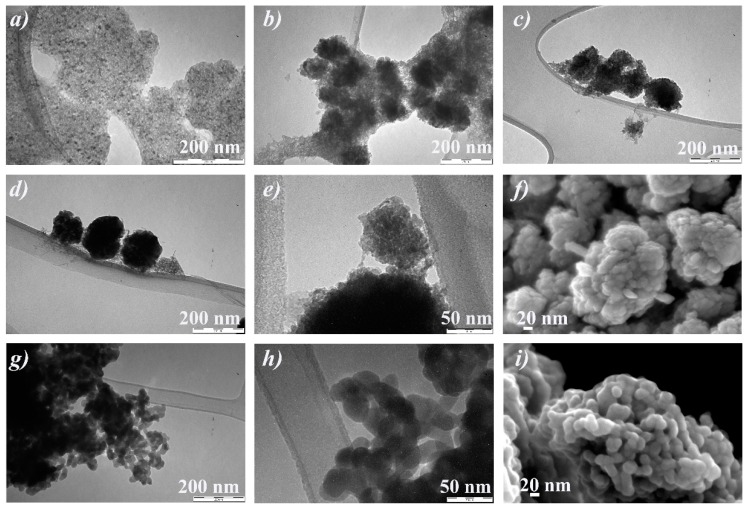
TEM images of samples observed after aging in water at RT for the following times: (**a**) One week, (**b**) two weeks, (**c**) two months, (**d**,**e**) seven months. (**g**,**h**) TEM images of the sample calcined at 500 °C for 3 h. FE-SEM images of the samples: (**f**) After aging in water at RT for seven months, (**i**) calcined at 500 °C for 3 h.

TEM and FESEM images both of the samples aged for different times at RT and of the sample calcined at 500 °C for 3 h are reported in Figure 8. TEM images, referred to the sample aged for one week, reveal the presence of dark particles, with dimensions of about 10 nm, homogeneously dispersed in the amorphous ferrihydrite matrix (Figure 8a). The further evolution is shown in the sample aged two weeks (Figure 8b), where darker and denser aggregates are visible in the ferrihydrite matrix, reaching diameters of about 50–100 nm. With increasing time, such aggregates become larger and well defined (Figure 8c), and after seven months, they appear organized into dense and pseudo-spherical clusters (Figure 8d,e). From FESEM image the pseudo-spherical clusters are clearly visible (Figure 8f), and they are characterized by two distinct morphologies of the assembled particles: A spherical morphology, attributed to the residual ferrihydrite, and a rhombohedral one, that can be related to the hematite particles emerging on the surface of the aggregate as well. These observations confirm the hypothesis that the phase transformation from ferrihydrite to hematite is due to an oriented attachment growth, where the ferrihydrite NPs form aggregates, already visible after two weeks of aging, and becoming larger with time until they define a typical crystalline morphology of hematite [55].

TEM images of the sample calcined at 500 °C (Figure 8g,h), show that the hematite NPs formed by calcination in dry conditions seem to have plate-like morphologies, almost hexagonal, with dimensions between 25 and 35 nm. These samples differ from the aged ones because they do not show an aggregation nature, and besides the ferrihydrite matrix is never observed. The corresponding FESEM image (Figure 8i) confirms that the NPs have all similar dimensions, of about 30 nm. The described above mechanisms, at the base of the transformation from ferrihydrite to hematite according to the two procedures, can explain why the NPs produced by calcination is smaller and monodispersed in dimensions with respect to those obtained after aging in water. Actually, in dry conditions, the reorientation of the iron ions is very fast, and the mobility of the ions is definitely reduced so inducing a fast nucleation and successively inhibiting the growth of the particles themselves.

**Figure 9:**
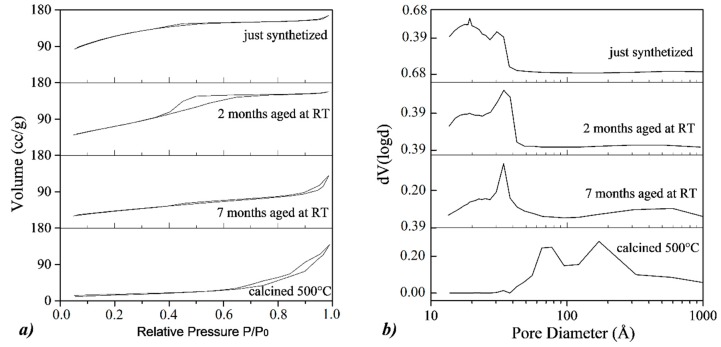
(**a**) N_2_ adsorption/desorption isotherms and (**b**) BJH distributions for the desorption branch of the isotherms.

The nitrogen adsorption measurements of the H_RT2_, H_RT7_ and H_500_ samples are shown in Figure 9 in comparison with their precursor sample (Fh_1M_). According to the IUPAC classification [50], the isotherm of the H_RT2_ sample can be related to isotherms of type IV, with the typical hysteresis loop of mesoporous materials, which is associated with capillary condensation taking place in mesopores. The isotherm of the H_RT7_ sample results comparable with type II, characteristic of macroporous materials, having a type H4 hysteresis loop, which can be associated with spheres composed of ordered mesopores. Finally, the isotherm of the H_500_ sample results comparable with type II too, but having a type H3 hysteresis loop, which is associated with non-rigid aggregates of plate-like particles forming slit-like pores.

Regarding the BJH distributions, the samples aged at RT show the presence of narrow mesopores (<4 nm), that can be related to the residual ferrihydrite, and macropores (20–100 nm), particularly evident in the H_RT7_ sample, and for this reasons, related to the formation of hematite. On the contrary, in the calcined H_500_ sample, the typical mesopores observed in the ferrihydrite samples are absent, while mesopores peaked at around 8 nm and 30 nm are observed, associated with the hematite phase. In Table 3, the summaries of the BET and BJH desorption analysis, in terms of surface area values and pore diameters for aged and calcined samples, are reported. It is possible to note that, increasing the aging time, BET values gradually reduce from H_RT2_ to H_RT7_ sample, ranging from 263 m^2^/g to 154 m^2^/g, respectively. This trend reflects the results discussed for the BJH desorption curves, so that the BET surface area of 154 m^2^/g measured for H_RT7_ sample can be attributable not only to hematite, but also to a residual content of ferrihydrite. On the contrary, for the H_500_ sample, a BET surface area of 57 m^2^/g is observed, and it is attributable only to the hematite phase.

**Figure 10:**
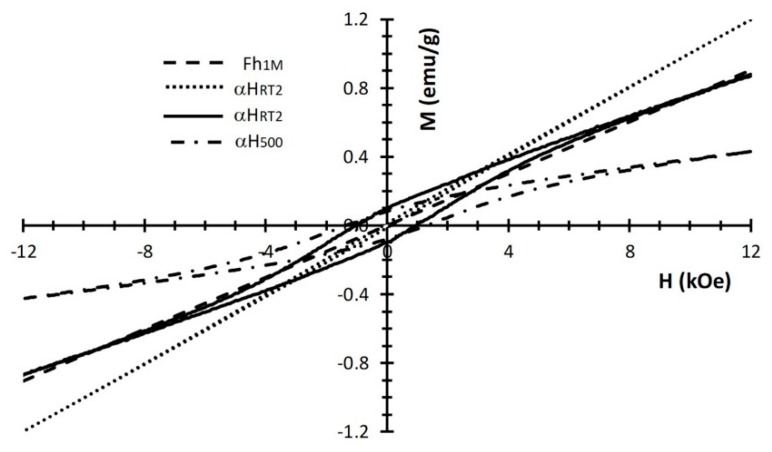
Hysteresis loops of different samples synthetized from 1M concentration of the aqueous ferric chloride solution: As synthetized (Fh_1M_), aged two months at RT (H_RT2_), aged seven months at RT (H_RT7_), and calcined at 500 °C (H_500_).

In Figure 10, the magnetization curves for the samples with different aging times or with heat treatment are reported. The initial increase of magnetic susceptibility after two months aging (H_RT2_) indicates that the average particle magnetic moment is increasing as a consequence of aggregation. In ref. [59] a similar and even more dramatic result, although under substantial different aging conditions, is observed and attributed to dipolar interaction between magnetic particles. A slight appearance of hysteresis is consistent with the formation of the hematite phase [55], as detected by the XRD patterns. At higher aging times (H_RT7_), the magnetization decreases as the abundance of hematite prevails. Consistently, magnetic hysteresis is more pronounced. This trend is confirmed for the sample calcined at 500 °C (H_500_), when the ferrihydrite disappears, and only hematite is present. Notice that also the shape magnetic anisotropy, as well as other morphology details, contributes to the higher observed coercivity of this pure α-Fe_2_O_3_ sample [12].

## 4. Conclusions

Iron oxide nanoparticles are very promising, among nanomaterials, because of their versatility in several application fields. However, for real-world applications, the synthesis of iron(III) oxide NPs should be based on readily available, inexpensive and non-toxic precursors, as well as simple synthetic methods, hopefully without intermediate steps limiting the production yield of the whole process.

The present study shows that pure 2-line ferrihydrite NPs can be synthesized by means of an innovative, time and energy-saving method, characterized by a very high production yield, based on an ion exchange process. The characterization analyses reveal that higher reagents concentrations produce smaller NPs, due to a higher degree of supersaturation and to a greater nucleation rate. The 2-line ferrihydrite NPs, organized in quite ordered and microporous/mesoporous aggregates, are spherically shaped and monodispersed, with size dimensions of 2–3 nm, and a BET surface area up to 421 m^2^/g, values higher than those previously reported in the literature for ferrihydrite samples.

The 2-line ferrihydrite NPs are then used as a precursor to produce α-hematite NPs, by following two different procedures: Aging in the water at room temperatures and calcination in dry conditions at different times and temperatures. After seven months of aging in water, rhombohedral particles of α-hematite, with average dimensions of about 50 nm, are observed; they assembled in pseudo-spherical mesoporous aggregates, but still containing residual ferrihydrite NPs. A corresponding BET surface area of 154 m^2^/g results probably affected by the ferrihydrite contribution. On the contrary, following calcination at a temperature of ≥500 °C, pure α-hematite NPs are obtained. Such NPs, monodispersed and with dimensions of about 30 nm, show a hexagonal plate-like morphology and a BET surface area of 57 m^2^/g. The results obtained by the characterization study reported in the present paper, help to describe the different mechanisms occurring in water or in dry conditions, at the base of the transformation from ferrihydrite into hematite. Actually, during aging in water, the transformation can occur by a gradual oriented attachment of the ferrihydrite NPs that, through dehydration and reorientation of iron ions, induces the crystallization of α-hematite. On the contrary, the calcination in dry conditions induces the ferrihydrite NPs to dehydrate and to aggregate with increasing temperature but, because of the restricted mobility of ions, the reorientation of iron ions occurs only at 500 °C, where the diffusion processes are enhanced.

The rapidity and the operating conditions of this novel synthetic route represent a cost-effective possibility to produce ferrihydrite and hematite nanoparticles on a large scale, allowing a more extensive application in several fields.

## 5. Patents

On the innovative synthesis method of 2-line ferrihydrite, which is based on an ion exchange process, it is pending an Italian national patent.

## Figures and Tables

**Table 1:** Chloride concentration (CC), the yield of the production (Y) and pH values during the synthesis, at different times t from the beginning of the synthesis and for different reagents concentrations: 0.1 M, 0.5 M, and 1 M.

	Fh_0.1M_			Fh_0.5M_			Fh_1M_		
t (minutes)	CC (mg/L)	Y (%)	pH	CC (mg/L)	Y (%)	pH	CC (mg/L)	Y (%)	pH
0	10,635	-	3	53,175	-	3	106,350	-	3
0.5	323	97.0	5	352	99.3	5	502	99.5	5
1	98	99.1	6	107	99.8	6	190	99.8	6
3	27	99.7	7	40	99.9	7	55	99.9	7
5	23	99.8	7	32	99.9	7	39	99.9	7
15	23	99.8	7	23	99.9	7	29	99.9	7
30	19	99.8	7	23	99.9	7	26	99.9	7
60	19	99.8	7	20	99.9	7	22	99.9	7

**Table 2:** Specific surface areas obtained by the Brunauer Emmett-Teller (BET) equation fit for N_2_ gas adsorption isotherms of the Fh samples, synthesized from different reagent concentrations and times of synthesis: 0.1 M, 0.5 M, 1 M and 1 M fast. Pore diameters and pore volume from BJH analysis are reported too.

Sample	BJH Pore Diameter (nm)	BJH Pore Volume (cc/g)	BET Surface Area (m^2^/g)
Fh_0.1M_	3.4	0.253	283
Fh_0.5M_	3.0	0.236	305
Fh_1M_	1.5	0.238	421
Fh_1M fast_	1.5	0.144	327

**Table 3:** Specific surface areas obtained by the BET equation fit for N_2_ gas adsorption isotherms of the samples of hematite obtained from two and seven months of aging at RT and from calcination at 500 °C. Pore diameters and pore volume from BJH analysis are also reported.

Sample	BJH Pore Diameter (nm)	BJH Pore Volume (cc/g)	BET Surface Area (m^2^/g)
H_RT2_	2.9	0.261	263
H_RT7_	3.5	0.200	154
H_500_	6.5	0.221	57

## Supplementary Materials

The following are available online at https://www.mdpi.com/2079-4991/10/2/323/s1, Figure S1: Chloride concentration (CC) values versus times during the exchange process, in relation to different initial chloride concentrations: (**a**) 0.1M, (**b**) 0.5 M and (**c**) 1 M., Figure S2: TG-DTA analysis at a heating rate of 10 °C/min of lyophilized powder from Fh_1M_ sample, Figure S3: FESEM micrographs obtained on standard stabs (suspension concentration of 10 g/L), at 200kX magnification for different samples: (**a**) Fh_0.1M_, (**b**) Fh_0.5M_, (**c**) Fh_1M_, (**d**) Fh_1Mfast_, Figure S4: ATR-FTIR spectra from 400 to 4000 cm^−1^ of the samples: just synthetized (Fh_1M_), aged two months at RT (H_RT2_), aged seven months at RT (H_RT7_) and calcined at 500 °C (H_500_).
